# Analyzing User Reviews of the First Digital Contraceptive: Mixed Methods Study

**DOI:** 10.2196/47131

**Published:** 2023-11-14

**Authors:** Marianela Ciolfi Felice, Marie Louise Juul Søndergaard, Madeline Balaam

**Affiliations:** 1 Division of Media Technology and Interaction Design KTH Royal Institute of Technology Stockholm Sweden; 2 Institute of Design AHO Oslo School of Architecture and Design Oslo Norway

**Keywords:** digital contraception, reproductive health, digital health, women’s health, intimate health, computational methods, natural language processing, NLP, user experience, health informatics

## Abstract

**Background:**

People in Western countries are increasingly rejecting hormone-based birth control and expressing a preference for hormone-free methods. Digital contraceptives have emerged as nonhormonal medical devices that make use of self-tracked data and algorithms to find a user’s fertile window. However, there is little knowledge about how people experience this seemingly new form of contraception, whose failure may result in unwanted pregnancies, high health risks, and life-changing consequences. As digital contraception becomes more widely adopted, examining its user experience is crucial to inform the design of technologies that not only are medically effective but also meet users’ preferences and needs.

**Objective:**

We examined the user experience offered by Natural Cycles—the first digital contraceptive—through an analysis of app reviews written by its users worldwide.

**Methods:**

We conducted a mixed methods analysis of 3265 publicly available reviews written in English by users of Natural Cycles on the Google Play Store. We combined computational and human techniques, namely, topic modeling and reflexive thematic analysis.

**Results:**

For some users of digital contraception, the hormone-free aspect of the experience can be more salient than its digital aspect. Cultivating self-knowledge through the use of the technology can, in turn, feel empowering. Users also pointed to an algorithmic component that allows for increased accuracy over time as long as user diligence is applied. The interactivity of the digital contraceptive supports mutual learning and is experienced as agential and rewarding. Finally, a digital contraceptive can facilitate sharing the burden of contraceptive practices or highlight single-sided responsibilities while creating points of friction in the required daily routines.

**Conclusions:**

Digital contraception is experienced by users as a *tamed natural* approach—a natural method contained and regulated by science and technology. This means that users can experience a method based on a digital product as “natural,” which positions digital contraceptives as a suitable option for people looking for evidence-based nonhormonal contraceptive methods. We point to interactivity as core to the user experience and highlight that a digital contraceptive might allow for collaboration between partners around contraceptive practices and responsibilities. We note that the user diligence required for the digital contraceptive to provide accurate and frequent data is sometimes not enough. Future research could look at designing (and redesigning) digital contraceptives with primary users and intimate partners, enhancing the experience of tamed naturalness; exploring how trust fluctuates among involved actors and in interactions with the technology; and, ultimately, designing more inclusive approaches to digital contraception.

## Introduction

### Background

#### Overview

With people in Western countries increasingly rejecting hormone-based contraceptive methods [1], digital contraception is emerging as a nonhormonal option. Digital contraceptives are medical devices designed to identify a menstruator’s fertile window to prevent pregnancy [2,3]. Although considered novel, digital contraceptives take on older technologies such as fertility awareness–based methods, which consist of the long-term tracking of signs of fertility to pinpoint ovulation, including the length of the menstrual cycle, patterns in body temperature, and changes in cervical mucus and position. Similar to all contraceptives, if a digital contraceptive fails or a user fails to use it correctly, it can result in an unintended pregnancy. This, in turn, implies high risks to health and potential life-changing consequences, especially where abortion is illegal or difficult to access [4-6]. Even though 99% of US-based women have used some form of contraception for an average of 3 decades [7], there is little knowledge [8,9] on how users experience this seemingly new form of contraception and what it offers to them beyond medical effectiveness. Shedding light on this dimension becomes particularly urgent when considering that the tracking performed in digital contraception constitutes a long-term project, which differs from other personal health contexts where short-term use predominates [10]. Thus, as digital contraception becomes more widely adopted, examining its user experience is crucial to inform the design of these intimate self-tracking technologies in a way that continues to meet users’ preferences and needs over time.

#### Intimate Self-Tracking Technologies in Contraception

Intimate self-tracking technologies have increasingly received attention in human-computer interaction in recent years, with a clear focus on the context of menstruation [11-18] and conception [19-22]. A smaller but growing body of work has started to study the use of intimate self-tracking technologies in the space of contraception. Mangone et al [23] surveyed the landscape of low-cost apps in the United States that offered some type of support for pregnancy prevention. They found various functionalities, spanning fertility tracking, birth control reminders, and sex and reproductive health information. However, they noted that most apps missed opportunities to provide users with evidence-based techniques and information on contraception. Similar findings were reported by Starling et al [24], who surveyed women in the United States who had used a fertility app with the goal of preventing pregnancy (or intended to use one in the future) and found that they wanted apps to be based on scientific evidence and capable of identifying their own fertile window. The authors warned that most of the apps mentioned in the survey were not designed for contraception (ie, users were appropriating [25] them for this purpose). More recently, the qualitative study by Algera [26] in the Netherlands involved 17 people who were using a variety of intimate self-tracking technologies with the purpose of avoiding pregnancy (regardless of whether these technologies were digital contraceptives or not). This study highlighted how self-knowledge can come as a result of the tracking augmenting users’ embodied experiences. Although these works provide a valuable foundation, they do not explicitly examine the experiences of users of a digital contraceptive. The most significant explorations in this particular space can be found in the studies by Park et al [8] and Chen [9], both of which are based on Natural Cycles. We expand on these 2 cases in the following section after providing the necessary background on this digital contraceptive.

#### Natural Cycles

Natural Cycles is a subscription-based digital method of contraception and pregnancy planning [2] and the first to be certified in the European Union and cleared in the United States to market as a software application for contraception [27,28]. Using this medical device requires a 2-decimal digital thermometer (or the Oura Ring) and the Natural Cycles app, where the user inputs their basal body temperature (BBT) and menstruation dates. Users can choose (and switch) between 4 modes: NC° Birth Control, NC° Plan Pregnancy, NC° Follow Pregnancy, and the newly added NC° Recovery (after pregnancy loss)—with the most used being the Birth Control mode [29]. When starting to use it in Birth Control mode, the app marks most days as potentially fertile (“red days”) and warns the user to use an additional form of contraception [30]. By tracking data over the course of several menstrual cycles, the algorithm develops an increasingly accurate identification of the user’s fertile window, indicating fewer red days and more “green days” [30]. Users need to measure their temperature as soon as they wake up, while still in bed [31]. To maximize the number of green days, users are recommended to measure their temperature at least 5 times a week [31]. In addition, it is advised to wake up at approximately the same time every day (−2 hours to +2 hours) [31]. According to Natural Cycles’ own medical evidence [32-35], users will experience a 98% effectiveness with perfect use (which accounts for unintended pregnancies happening as a result of condoms breaking or the method giving a wrong green day) and a 93% effectiveness with typical use (which also includes cases of unprotected sex on a red day). Natural Cycles has also funded studies based on their users’ self-tracked data (most of whom lived in Sweden, the United Kingdom, and the United States), analyzing the quantitative characteristics of users’ menstrual cycles [36] and examining the time to pregnancy for users in the Plan Pregnancy mode [37]. Recent company-funded (but independently run) interview studies with users of the Plan Pregnancy mode in the United Kingdom [21,22] showed that the app supported self-knowledge, which aided users’ preconception and conception work and health care while also reproducing highly gendered roles in this context [21]. In total, 2 studies have looked instead at the contraceptive experiences of Natural Cycles users in Birth Control mode [8,9]. The Master’s thesis by Chen [9] contains 12 interviews from 2017, before the product was cleared in the United States. The analysis by Chen [9] emphasized that the app supported users (all cisgender women in Sweden) in gaining knowledge about their own bodies and fertility. It is also clear from the study by Chen [9] that avoiding hormonal contraceptive methods and biomedical interventions was a key motivation for participants to use a digital product. More recently, Park et al [8] conducted design workshops with 14 users (mostly situated in Scandinavia and other parts of Europe). The analysis highlighted the ambivalent feelings that participants had developed toward their digital contraceptive over time. Arguing that these mixed feelings are unavoidable in the interaction between humans and technology, the authors offered strategies for designing intimate self-tracking technologies using ambivalence as a generative resource. Although these studies provide valuable entry points for understanding the user experience of digital contraceptives, a broader examination of the experiences of digital contraceptive users worldwide is, to our knowledge, still missing.

#### User Reviews as a Source of User Experience Insights

App reviews can help identify which aspects of a product users deem important for others to know [38] and constitute a rich source of information on how a technology is used in the wild [39]. Pagano and Maalej [40] have demonstrated that user reviews are not just used by developers as crowdsourced system requirements but that they affect the market and the community. Moreover, Simonson and Rosen [38] have shown that people use and trust reviews as a fundamental feature in their decision-making process. In addition, user reviews have been successfully used by researchers to understand users’ experiences with digital technologies [39-41], including the contexts of health and well-being [15,42-48]. Given this background, we argue that an analysis of user reviews of Natural Cycles can provide an entry point to their experiences with digital contraception.

#### Combining Computational and Human Analysis Techniques

Computational techniques can help researchers interpret large textual data sets by providing, for instance, unsupervised detection of topics using models. Concannon et al [49] and Gill et al [50] have argued for the use of computational techniques as an empirically grounded entry point for qualitative analysis of textual data and showcased the strength of using, among other techniques, topic modeling and in-depth human analysis. Topic modeling has the potential to detect unexpected topics that researchers can then analyze. Its strength lies in finding relationships among words, allowing for the generation of relevant insights that might go unnoticed when focusing on frequency or rarity. Probabilistic topic modeling [51-53] assumes that a document can be described as a mixture of topics and that a topic can be modeled as a distribution of probabilities over words. Latent Dirichlet allocation (LDA) [51] is one of the most established techniques. LDA assumes that the corpus contains *k* topics (user-specified parameter) and that each document talks about these topics to a certain extent. When using it with short texts, it is important to let topics vary in importance and, in particular, to allow for “favoring” of a few topics per document [53,54]. This can be achieved by manually setting hyperparameters [55,56] or using hyperparameter optimization [49,54].

### Goal of This Study

We wanted to understand the user experience of digital contraception to better inform the design of digital contraceptives. Unpacking this user experience involves going beyond medical effectiveness, studying why users choose and keep using digital contraception and the challenges they face. This goal motivated us to analyze user reviews of the Natural Cycles app, the first digital contraceptive. By focusing on publicly available data generated by users, we were able to incorporate a large number of users’ voices worldwide and investigate which experiences people chose to share with other potential users or the company itself. By doing so, our work builds on the work by Ng et al [57], which highlights how less dominant knowledge can become socially intelligible and influential within decision-making processes. Our work contributes a mixed methods analysis of 3265 user reviews of Natural Cycles on the Google Play Store.

## Methods

### Overview

Given the large number of user reviews, we combined human and computational methods, including topic modeling and thematic analysis. More specifically, we used LDA as a starting point for human analysis (similar to the studies by Concannon et al [49] and Gill et al [50]) as this technique can bring to the foreground topics that may not be so prevalent in terms of coverage but that reveal an interesting aspect of users’ experiences.

### Data Collection and Preprocessing

The first author wrote a script that uses an open-source scraper [58] to download all the reviews of the Natural Cycles app on the Google Play Store and a Python-based pipeline that uses the open-source libraries spaCy [59] (Explosion AI) and Gensim [60] (RARE Technologies Ltd) to preprocess the data. We did not include Apple App Store reviews because of a restriction imposed by this store under which only the last 50 reviews of an app could be fetched. We focused on English-based reviews (≥6 characters) as this is a shared language that the 3 authors speak fluently and feel confident in interpreting. Data cleaning included transforming the text to lower case and removing numbers and stop words.

### Data Analysis

#### Topic Modeling

Our pipeline uses the Little MALLET Wrapper [61] (a Python-based wrapper for the MALLET Java implementation of LDA [62]) to perform topic modeling. To choose *k*, the number of topics in the model, we used Gensim’s implementation of the pipeline by Röder et al [63] to compute the coherence score of different possible LDA MALLET models (coherence scores are an established way of measuring the quality of a topic model as they correlate with higher human interpretability). We ran the comparison with *k* ranging from 4 to 24, discarding larger values as they did not increase the score enough to compensate for the human labor of interpreting and labeling topics. We ran LDA with a hyperparameter optimization interval of 10 and 100 sampling iterations. To interpret and name the topics, we started exploring the model, becoming familiar with the top 15 keywords of each topic. For each topic, the first author read the 10 most representative reviews (ie, the documents with the highest probability for a certain topic). We combined the topic keywords with the most representative reviews in a process guided by the following questions: (1) What do they talk about and how do they use the topic keywords? (2) On which grounds are these reviews assessing Natural Cycles? This allowed us to interpret the model based on the aspect of the product or experience on which the topic keywords and representative reviews centered, which in turn helped us name and summarize the topics, refining our interpretation in discussions among the authors.

#### Thematic Analysis

Different types of thematic analysis have been combined with topic modeling to interpret topics and patterns across them more deeply [46,47,56,64-68]. In this study, we used reflexive thematic analysis [69,70] as this approach is suited for addressing research questions regarding people’s experiences. In particular, it recognizes the situated and partial nature of coding, requiring researchers to reflect upon and acknowledge the role that they play to ensure rigor in qualitative analysis [71]. Thus, we offer a reflection to contextualize our analytical process. We, the authors, are cisgender women who consider themselves intersectional feminists [72]; were raised in Argentina, Denmark, and England; and are affiliated with academic institutions in Sweden and Norway. We have varying degrees of firsthand experience with menstrual cycles, contraception, pregnancy, and intimate self-tracking. As interaction designers and researchers, we build upon frameworks and agendas, including Feminist HCI [73], data feminism [74], and antiessentialist woman-centered design [75,76], underpinned by our feminist epistemological commitments, especially the situated knowledges by Haraway [77]. Our identities and our theoretical and pragmatic influences prompted attention to how inequalities and power relations—including norms and responsibilities surrounding contraception—are mediated and reconfigured by digital technology and how the body and data about the body are coproduced.

We constructed themes across topics generated by the LDA to best capture what the review authors considered to be most important about their experiences with Natural Cycles as a digital contraceptive. First, the first author inductively coded the 10 most representative reviews for each of the 12 topics. In conversations with the coauthors, we constructed and iteratively refined 5 themes from the coded data. Once the themes were stable, the first author applied a deductive analysis step over a larger set of representative reviews for each topic to further develop our analytical take on the roles that a digital contraceptive played for the review authors.

### Ethical Considerations

Our study was approved by the Swedish Ethical Review Authority for research under diary 2021-04517. The study is based on publicly available data that review authors made public in concordance with the Google Play Store’s privacy policy for ratings and reviews [78]. We accessed the reviews in compliance with the Google Play Store’s terms of service [79] using a publicly available scraper [58]. Even though the reviews were public, to further protect the review authors, we did not store any identifying information about them, such as their username. Our data set only contained the following fields: review date, score given to the app, review title, review text, app version, number of thumbs-up the review received, and the Play Store language. In addition, we kept our data set offline in a secure, password-protected device in a locked cabinet. Finally, to the best of our knowledge, the review fragments we selected to be in this manuscript do not risk reverse identification. Our study is not funded by Natural Cycles.

## Results

### Overview

Our scraper script obtained 5247 reviews posted up to September 2021. We detected 62.23% (3265/5247) of the reviews in the initial data set that were at least 6 characters long and written in English. These were, on average, 183.48 (median 132, SD 172.25) characters long, or 34.28 (median 25, SD 32.14) words long. Most reviews (2468/3265, 75.59%) gave 5 stars to the app, 12.53% (409/3265) gave it 4 stars, 6.71% (219/3265) were 1-star reviews, and the rest were 3-star (89/3265, 2.73%) and 2-star (80/3265, 2.45%) reviews. The vast majority (3114/3265, 95.38%) of review authors used their Android phones in English. The rest (151/3265, 4.62%) were divided among 27 other languages, with only slightly more users using it in Swedish (26/3265, 0.8% of users), French (19/3265, 0.58%), Dutch (17/3265, 0.52%), and Portuguese (14/3265, 0.43%) and <10 users in each of the other 23 languages (75/3265, 2.3%). The earliest reviews were from 2014, and most (1873/3265, 57.37%) were posted between 2017 and 2018.

### Resulting Topic Model

**Figure 1:**
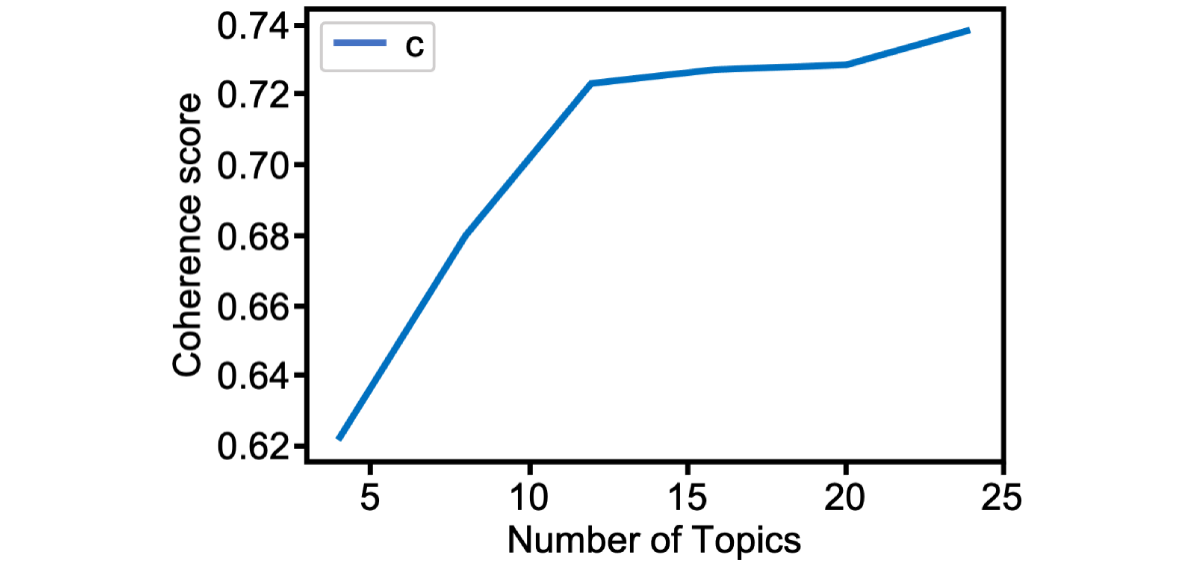
Coherence scores for latent Dirichlet allocation models going from k=4 to k=24, in steps of 4.

We found that *k=12* yielded the best results in terms of granularity (Figure 1). We present the 12 topics that comprise the topic model in Table 1. The table shows the name we assigned to each topic and the topic keywords. Our interpretation includes the aspect of the product or the experience that each topic related to and a short descriptive summary. Except for the topic keywords, which are an LDA output, the other columns constitute the qualitative effort necessary to make sense of the topic model.

When analyzing the topics quantitatively, we see that the most prevalent is topic 10, *Satisfied with app-based contraception* (23.61% probability), followed by topic 0, *Natural contraception* (18.04%), and topic 8, *Body or algorithm* (10.92%; Table 1). The prevalence represents the topic’s coverage of the corpus and was calculated by weighing the topic probability for each document against document length and then normalizing with respect to corpus length.

**Table 1:** Our interpretation of the topic model: named topics with keywords, prevalence, main aspect in the reviews, and summary, ordered by prevalence (descending).

Number	Topic name	Topic keywords	Prevalence	Aspect	Summary
10	Satisfied with app-based contraception	easy_use, great_app, love, app, work, accurate, great, love_app, cycle, help, far, body, helpful, highly_recommend, like	0.2361	App design	Positive assessment of the concept and its execution via an app
0	Natural contraception	cycle, contraception, body, love_app, know_body, birth_control, pill, hormone, year, long, woman, easy, contraceptive, feel, happy	0.1804	Concept	Natural contraceptive that allows users to be in touch with their bodies
8	Body or algorithm	day, app, month, green_day, period, year, pregnant, start, right, great, week, good, expect, test, predict	0.1092	Different trusts at stake	Deciding when to trust the algorithm and when to trust one’s own body
7	Frustrating routines	thermometer, app, temp, temperature, morning, wake, time, reminder, measure, update, problem, sleep, phone, notification, thing	0.0995	Product and service design limitations	Critical assessment of product and service design
3	Why pay for it?	pay, app, free, subscription, money, want, try, month, email, buy, charge, refund, expensive, account, service	0.0929	Business model	Negative assessment of the business model
1	Effective and versatile	pregnancy, baby, birth_control, plan, prevent_pregnancy, fertile, easy, safe, get_pregnant, pregnant, conceive, chart, start, help, switch	0.0608	Result	Positive assessment of the product and its versatility based on results over time
4	Liberating contraception	control, feel, find, day, helpful, recommend, hormonal_birth_control, pattern, big, extra, allow, love, health, process, great	0.0523	Conceptual consequence	Liberating alternative to hormonal contraception that supports finding oneself, going beyond contraception goals
11	Data needs	datum, like, option, add, note, day, graph, symptom, cycle, input, record, different, pain, thing, cervical_fluid	0.0521	Difference (from other digital methods) and unmet data needs	Data needs (limitations and opportunities) and differences from similar digital products
2	No side effects contraception	pill, iud, come, pretty, cause, control_method, work, people, cycle_app, implant, fertile, depression, try, menstrual, decision	0.0367	Difference (from other nondigital methods)	Uniqueness as contraceptive (old technique [BBT^a^] with a novel digital approach and no side effects)
5	Cost of knowledge	help_understand, different, algorithm, shift, love_natural, protection, try_baby, successfully, detailed, self, green_day, struggle, symptom, sex, phase	0.0313	Perception of a process with a cost	The algorithm rewards diligence and patience with increasing knowledge and accuracy
9	Trust or distrust in BBT method	system, purchase, method, commit, price, win, risk, temperature, suggest, app_take, year, basal_thermometer, good_app, contraception, sure	0.0248	Method trustworthiness	Trusting or not trusting the BBT method behind the proprietary algorithm
6	Empowering yet normative	woman, life, long_time, hormonal_birth, decide, young, hope, regulate, doesn_work, teach, community, fit, base, accurate_app, family_planning	0.0238	Societal (yet gendered) potential	Self-understanding scaffolds self-compassion, and this is empowering for (some) women

^a^BBT: basal body temperature.

### Constructed Themes

#### Overview

Table 2 shows a summary of the themes. There is no one-to-one mapping between topics and themes as a theme is not simply a collection of topics. Instead, we indicate in the table which are the topics that contributed the most to each theme. Certain topics are more present than others because of their stronger links to users’ contraception experiences.

**Table 2:** Summary of themes.

Theme	Characteristics	Topics represented
Hormone-free contraception: a certain type of natural	A dichotomy between “good” and “bad” hormones (natural vs artificial) motivates the choice of using a DC^a^. A DC is perceived as an alternative method in which its hormone-free aspect predominates over its digital aspect.	0 (natural contraception); 2 (no side effects contraception)
Empowering contraception: (Re)Turning to a normal self via self-control	The concept of a “normal self” that can be reached by using a DC and, in particular, by first cultivating self-knowledge. A normal self is not just in touch with but also in control of itself—which feels empowering.	4 (liberating contraception); 6 (empowering yet normative)
Algorithmic contraception: accurate if “used properly”	Being based on personal, quantitative data, the DC is seen as trustworthy and scientific as it becomes progressively accurate over time but only if user diligence is applied—excluding certain types of bodies and life situations.	5 (cost of knowledge)
(Inter)active contraception: rewarding and agential	The DC is seen as interactive—not only does it respond to input data, it also takes the initiative, which contributes to its trustworthiness and perceived support. Expectations around mutual learning.	8 (body or algorithm); 10 (satisfied with app-based contraception); 11 (data needs)
Collaborative contraception: shareable responsibility and new frictions	Interpersonal, collaborative aspects of contraception. The DC facilitates sharing experiences and burden (or highlights single-sided responsibility) while creating points of friction in the new routines.	2 (no side effects contraception); 7 (frustrating routines)

^a^DC: digital contraceptive.

#### “Hormone-Free” Contraception: A Certain Type of Natural (Theme 1)

This theme captures a key conceptualization in users’ narratives: the notion that there are “good” and “bad” hormones. “Good” hormones are produced by the body during the menstrual cycle:

Having used various forms of contraception over the last 30 years, my hormones have been suppressed and this has led to various health issues. ...My hor[m]ones seem to be more balanced now. I feel natural and free!

These hormones are regarded as “natural,” and the reviews widely show a positive judgment of that which is natural. “Bad” hormones, in contrast, are the synthetic hormones consumed through hormonal contraceptive methods such as contraceptive pills, implants, intrauterine devices, or injectables. These hormones are seen as “artificial,” which is perceived by users as undesirable.

Upholding this dichotomy affects how some users choose and judge digital contraception. The reviews often valued this digital contraceptive in direct comparison with hormonal contraceptive methods:

...much prefer it to bombarding my body with hormones!

...going natural feels so much better than pumping hormones into your body.

These reviews pose synthetic hormones as “bad” from a conceptual standpoint as they constitute an external source that controls the body (topic 0, *Natural contraception*). Other users described specific side effects of hormonal methods, opposing synthetic hormones from a practical stance instead (topic 2, *No side effects contraception*):

After suffering severe depression with the pill and constant bleeding with the jaydess IUD I was starting to hate my body for being so sensitive. Now I’ve found a way to work with my body instead of fighting against it.

Moreover, most topics capture that Natural Cycles was explicitly defined by users as a natural (0, 1, 2, 4, 5, 6, 9, 10, and 11) or noninvasive contraceptive method (0 and 2), which users highly valued. In addition, Natural Cycles was literally framed as an “alternative” to hormonal methods in 3.77% (123/3265) of the reviews. Interestingly, Natural Cycles is seen as a certain type of natural, a hormone-free one:

This app gives women the freedom of birth control without disrupting her hormonal balance. Wonderfully natural!

Some users valued the nonhormonal aspect of the product over its capability to give them enough green days:

So far I’m only 8 months in so it’s still getting to know my cycle, which means there are still quite a few days in the month where I need to use protection during sex. However, I love this idea that I don’t have to put hormones or have invasive things put in my body in order to avoid pregnancy!

Mentions of science or a scientific community behind the digital contraceptive arose in the reviews, implicitly posed as adding credibility to a “natural” method:

After 15 years of hormones, so glad science has finally developed a natural alternative.

Moreover, some users explicitly posed that the science is natural:

Managing my fertility the way I want to, the natural (science) way!

It is reliable, perfectly natural, and it’s more than just an app—here is an entire scientific community to provide answers and support to a vast user community.

The medical framing of Natural Cycles contributes to its trustworthiness in users’ eyes, being certified as a medical device “by real, regulatory agencies”:

It’s great peace of mind that it has been certified for use as a contraceptive method in some countries.

However, a few users implied a tension between nature and science when clarifying that Natural Cycles is “natural, but backed by science” and more subtly between natural and safe:

It’s an amazing way for women to get to know their bodies better and promote safe practices in a natural way.

In summary, Natural Cycles is seen as “natural” because it is based on a fertility awareness–based method and, thus, is nonhormonal. This theme captures how the experience of digital contraception can be more about what the product does not do rather than about its features. It shows that, in digital contraception, the hormone-free aspect of the experience can be more salient than its digital aspect.

#### Empowering Contraception: (Re)Turning to a Normal Self via Self-Control (Theme 2)

This theme captures the notion of a “normal self” and the path to it. The reviews suggested that users believe in the existence of a normal self that is good to discover or rediscover—if it was, for example, masked by or hidden behind hormonal contraception:

I love that I’m really getting to know my body. After using the pill for 6 years, my hormones were all over the place. However since getting Natural Cycles, I am my normal self again. I really love that my body is doing exactly what it needs to be doing naturally.

This normal self is the “natural” self, the “free” self, one that can be in control, which makes users feel empowered and liberated:

...Finally there is an app which gives me control and allows me to learn more about my ‘normal’ and pill free self ...

Taking ownership and understandi[n]g of [m]y own body back feels liberating.

This theme captures the idea that (re)turning to a normal self is a process that starts with noticing cyclical changes in bodily experiences, allowing users to understand their body and, through that, feel in touch with it, developing self-knowledge:

Fantastic way to learn more about your body! You start to notice certain regularity with everything, moods, energy levels. ...Recommend for all the women to reconnect with their bodies!

Self-knowledge can be a door to self-control—“This app has taught me so much about myself and has granted me the control over my own body that I have felt robbed of for years”—and to self-compassionate, dignified decisions:

...very enlightening to my understanding of how I ovulate. Keeping track of my symptoms and moods has helped me to better regulate and structure my life and be more compassionate with myself.

It helps me plan things in my life based on my changing energy level throughout the month... [I]t’s really changed my life and helps me make better decisions with my body & my partners. Every woman would benefit from this.

A key link in this theme is that of self-control as a path to a normal self:

I feel more myself every day instead of feeling so up and down and emotionally dull. Natural cycles is helping me to get to know my body and feel completely in control.

This (re)turning to a normal self also made some users leap from the personal to the societal and start thinking about the “liberation” or “empowerment” of “women”:

Love the information it provides about your own unique cycle, as well as general knowledge about female reproductive system. Great tool to monitor your fertility & empower women!!

Empowering. All women need this. Girls need to be taught this at a young age.

In summary, accounts within this theme go beyond being free from the side effects of synthetic hormones to actually acting upon this freedom, using it as a means toward, for example, a compassionate self-organization. Notions of control as self-ownership became salient in users’ experiences with the digital contraceptive. At a broader level, the potential empowerment or liberation of women comes up as a consequence of personal empowerment.

#### Algorithmic Contraception: Accurate If “Used Properly” (Theme 3)

Natural Cycles is based on an algorithm that uses personal quantitative data as input and provides increasingly accurate outputs over time. This reliance on quantitative data (referred to as “stats,” “math,” or “science” in the reviews) makes users regard it as scientific, which they in turn see as trustworthy. For example, a user explained the following:

The more you add value[s], the most accurate results and more green days. You can compare your data with the statistics and know you more. Also, it’s easy to use and they have great and serious documentation for knowing you and usin[g] the app correctly. For me, this is scientific.

In some reviews, technology appeared as a concrete means to implement “the science” (eg, “This isn’t a new method it’s just new tech”). Similarly, a user defined the uniqueness of Natural Cycles as an “old-school technique” (the BBT method) “with a modern approach” (an app and a dedicated algorithm).

Users highlighted the algorithm’s temporality, which is designed to become progressively accurate and trustworthy:

Easy to use and helpful learning phase builds confidence in the algorithm.

Many reviews explained that the algorithm needs time to “learn” the user’s cycle:

It does take a few months for the algorithm to get to know your cycle.

Moreover, users explained that the algorithm learning their cycle implies that it is capable of “accepting” its length to adapt the predictions. For example, one user struggled precisely with this:

My cycle is extremely regular but is longer than usual (38 days) and the app is slightly too slow at realising that this is not a mistake and that’s indeed how I function. I’m going onto the 3rd month using it and it still keeps predicting my ovulation and period on the wrong days.

There was a perception of a process that happens over time—typically months—revealing both users’ patience and expectations about this increasing accuracy:

The only reason I didn’t give it five stars is because it can be really difficult for the app to locate your ovulation day if you have highly irregular periods like I do. However, that being said, given enough cycles, it will get better at it and more accurate.

However, learning does not come without trouble as sometimes the algorithm’s output contradicts embodied feelings. Reactions to this are captured by topic 8, *Body or algorithm*. We see different ways of trusting competing, for instance, with people trusting their own bodies over the algorithm. For example, a user trusted their ovulation pain and double checked with other methods instead of accepting the algorithm’s output:

I’m always in pain when ovulating and I have double checked it with an LH test but when I log it on the app, it shows my ovulation was on another day. How can I change it so it will reflect my pain?

The reviews showed that users are aware that this learning requires something from them: user diligence in the form of careful, persistent labor. They often mentioned that the algorithm becomes more accurate if the user does “their part,” if they are “disciplined,” “committed,” and “dedicated” to measuring and logging their temperature. A few users did not seem to perceive “their part” as a burden at all:

...you don’t have to worry about managing anything, just give it the accurate data everyday, and it will do all the work for you.

Instead, other reviews mentioned the critical task that the user is in charge of:

Predictions are cautious and rely on your input. It’s up to you to identify when your temp might be off, so obviously its success will depend on how well you use it!

Have to be strict with remembering to check temp first thing as algorithm only as good as data you put in.

The reviews showed that, sometimes, user diligence is not enough, and some users pointed out that the product’s assumptions can be an insurmountable barrier to use. A recurrent barrier related to assumptions about sleeping patterns, which are affected by being a parent of small children or working night shifts. This includes the quantity of sleep and the regularity of waking-up time, which poses challenges in measuring the temperature:

Honestly, this app is only useful if you are a very specific person with a very specific set of circumstances. As someone who is self-employed with irregular sleeping patterns and inconsistent schedule, the algorithm was pretty much incapable of doing anything with my information because of the fluctuations in my measurements. For the 30-something crowd with regular 9-5’s, a stable relationship, and a calm social life, this is the app for you. But for me? After two years of trying to get this app to do anything with the measurements I put into it, I gave up. That isn’t to say it doesn’t work. For a very specific type of woman, it certainly does. But not all women have the same lives, interests, or goals. This app has literally no benefit for those of us who fall outside the “norm.”

Great if you’re on normal cycles. Not particularly helpful post-birth and breastfeeding.

For some reviewers, the app’s design and business model suggested certain types of users and data. For example, the reviews questioned assumptions related to monogamy (as some users would like to have the “opportunity to add multiple different contraception types on the same day to be more inclusive of poly relationships”), heterosexuality (“...this app is very heteronormative and does not take into consideration that some of us users are not in a relationship with a man”), and socioeconomic status (“My main complaint, however, is that by charging a substantive monthly fee to use the app, which in all likelihood American health insurance will never cover, you are keeping this resource out of the hands of low-income folks who could arguably benefit the most from it. I hope you will reconsider the pricing...”)*.*

In summary, this theme starts to give a glimpse of what a digital contraceptive actually “does” while highlighting the required user labor to be accurate, including assumptions of use and, as a consequence, exclusions—users reflect upon who can make “proper” use of the digital contraceptive given normative assumptions about bodies and lives that are not always compatible with theirs*.*

#### (Inter)Active Contraception: Rewarding and Agential (Theme 4)

On the one hand, being digital allows the product to store and respond to tracked data, providing personalized visualizations that make the experience of using the digital contraceptive rewarding for many users. On top of this, the product takes the initiative by sending in-app messages to communicate with the user, specifically to educate; motivate; and, in some cases, even “warn” them, which makes it be regarded also as agential.

Users widely appreciated the visualizations of their own data (in the form of graphs, calendars, and statistics), which play different roles—from saving users manual effort and motivating them to track more to facilitating the detection of patterns, the contrasting of app predictions and bodily experiences, and the cultivation of self-knowledge and being a token of the company’s credibility and trustworthiness. Reviews associated with topics 10 (*Satisfied with app-based contraception*) and 11 (*Data needs*) captured several of these roles:

I have been using fertility awareness for contraception for 10 years, and I love this app. It puts all the work I did manual charting into easy graphs.

This app provides you with a lot of information to understand your cycle and manage your fertility without a ton of record keeping and data crunching yourself. Just plunk in the temperature and see all the cool stats appear.

Going beyond functionality, users found it pleasurable to interact with the product, especially when they obtained an output that rewarded their diligence:

...it is really satisfying to input the data and see a result.

Our analysis suggests that a key role that in-app messages and visualizations play is that of improving self-knowledge. Self-knowledge is achieved through 2 mutually influencing aspects of the experience. On the one hand, users learn about their bodies when they start noticing their bodily experiences throughout the menstrual cycle. In addition, they also learn through feedforward from the app (eg, being notified of the first red day approaching) and feedback (eg, when it retrospectively adjusts the ovulation date based on new temperature data or results from luteinizing hormone tests). These interactions make users pay more attention to their bodies and contrast their embodied experiences with the digital data. Sometimes, the latter was used to confirm the former:

It feels great to have my ‘inklings’ confirmed as they arise.

I feel so in tune with my body and I’m learning to recognize EXACTLY when I am going to ovulate and menstruate...which is confirmed by the app.

Users also learned to detect deviant temperatures and appreciated being able to influence the algorithm:

It’s very clear and reminds you the importance of a deviating temperature so if your sleep is disturbed or you’ve been drinking that temp won’t count towards your algorithm. Thanks!!

Importantly, and contributing to the idea of the digital contraceptive as agential, the learning was often perceived as mutual. For example, some declared that they learned together with the digital contraceptive:

Have learnt to read my own bod[y’]s signs alongside the app.

Some users attributed intelligence to the algorithm, which justified its use of agency:

It’s so smart that it notices if your temperature is unusually high, and it doesn’t take it as a true measure.

In this theme, it is key that the digital contraceptive also takes the initiative via personalized in-app messages, which makes it be perceived as interactive and agential:

I also love how this app sends you messages along your journey. It’s really helpful and interactive which I love!

There are a myriad of ways in which app-initiated interactions unfold, including motivating the user to continue entering data to feed the algorithm and increase its accuracy (“This app does a good job motivating me to log my temperature regularly”); providing timely insights (“...full of helpful information about women’s health AND information about my specific cycle”); and triggering notifications when new predictions are ready, which some users saw as a token of accuracy and trustworthiness (“The app calculated exactly when my period was coming up with just a few hours difference with a notification. Pretty impressive!”). Interestingly, some users expected a reaction from the app when they ignored Natural Cycles’ instructions:

Strange thing is I did input unprotected sex on a red day but it didn’t give any message as a warning...Normally the app is quite messagey on certain things.

In summary, being digital does not simply mean having an algorithm manage data and calculations on behalf of the user. Being digital allows the digital contraceptive to be interactive, taking the initiative to communicate with the user, reassuring them, and playing a pedagogical and motivational role. This theme captures that app-initiated interactions help the user help the algorithm, nurturing perceptions (and expectations) of mutual learning—all of which contributes to users seeing the digital contraceptive as having agency and the interaction with it as rewarding.

#### Collaborative Contraception: Shareable Responsibility and New Frictions (Theme 5)

When a contraceptive becomes digital and interactive, with data potentially visualized on different devices and a routine to be maintained, new opportunities for others to be included in the experience arise. The reviews captured how using a digital contraceptive supports communication with different stakeholders. For some, having a partner following the cycle through access to the app improved communication:

Also love that my partner can be involved in the process; We both have the app (the account is linked). He always knows, and can help track, my cycle. ...We have found the added bonus of it being helpful for our communication.

For others, using a digital contraceptive helped the user “show” what was going on to their partner (“Also great for making it real to my partner!”) or to health professionals (“Having all the data on your cycle is helpful. Especially when i[’]ve gone to the doctor’s and they ask questions about your cycle”).

Some users also opened up about the topic with others:

One of the funny side effects of using this app (as opposed to hormones) is that I love telling people about it and sharing!

Some reviews also pointed to the need of a community to share progress and concerns (topic 6, *Empowering yet normative*).

The level of involvement of partners varied. Some partners took active roles in the use of the digital contraceptive:

I love how interactive it is on terms of keeping your partner in the loop too. He even shoves the thermometer in my mouth of I’m not listening to my alarm. Team work all the way....

My husband and I are both active participants in this process and he is more aware of my cycle and how it works. It’s no longer some great mystery.

Moreover, some partners were the ones initiating the measuring and inputting the data:

I’m not great at consistency and the thought of tracking my own fertility is overwhelming, but with this app my husband can download it too. Then he hands me the thermometer every morning and records my temperature for me.

Several users explicitly described the product as a means to share the burden of contraception in terms of effort (“It also helps share the burden of contraception with your partner as they can track your fertility with you and better understand what it means”) or costs (“Also, since you are going to use this with your partner it is only fair to share the costs”). In contrast, one user compared the digital contraceptive with the contraceptive pill, saying that, back then, they were “the only one in a relationship to have this responsibility” and that, with Natural Cycles, “I still have a responsibility with this app, but at least it is unobtrusive and extremely informative....” Similarly, several reviews depicted partners as passively affected by what users saw as design flaws—the sound and light of some thermometer models, which would wake up the partner, as captured in topic 7, *Frustrating routines*:

...I have to use my cellphone light to see the numbers in every morning, and I’m waking up my bf sometimes when I do this. Also, create the option to the thermometer to vibrate instead of making noise. It makes my partner angry sometimes when he wakes up because of the beeping.

At the same time, for some couples, being able to rely on a digital contraceptive brought new possibilities to their intimacy:

I’m so grateful for this app because up until I found it I felt like I had no chance of a normal sex life as I was unable to use an[y] form of contraception and my other half couldn’t get on with condoms. so this has change[d] my life!

In summary, this theme shows that a digital form of contraception can open up conversations and change users’ sex lives, fuel the need to share experiences with others, and configure a collaborative responsibility among partners—or highlight its absence.

## Discussion

Natural Cycles describes itself as *nonhormonal* birth control that pairs “the science” of the BBT method with an *algorithm* that learns the user’s cycle patterns without being *invasive* [80]. We offer a contextualization of our analysis in relation to relevant literature, discussing our results regarding the user experience of this digital contraceptive across these 3 components—nonhormonal, algorithmic, and noninvasive.

### Not Just Hormone Free: A Tamed Kind of Natural

Theme 1 captures that the experience of using a digital contraceptive is a special type of *natural*—a *hormone-free* natural—and that this experience can be more salient for some users than the digital experience. Theme 2 complements this with stories of self-knowledge acquired by merely noticing bodily changes previously masked by hormonal contraception. Interestingly, users attributed the improvements stemming from noticing to the digital contraceptive, whereas the digital contraceptive did not actually *do* anything in particular in that regard. In fact, the reviews showed that this conceptualization of the product as natural (with its positive judgment) can be formed even before using the product for a long time and getting a sense of its quality. In other words, a digital contraceptive does not have to “do” much at the beginning of the user journey when the main reason for using it is to have a nonhormonal option. The extent to which this method actually “works” as a stand-alone contraceptive (ie, providing enough green days in which another form of contraception is not needed) is, for some users, not the priority at the moment of writing a review. In other words, some value the absence of synthetic hormones (and the subsequent self-knowledge) more than the capability of the algorithm to give green days.

Our analysis shows that Natural Cycles is seen as a new, natural alternative to the default—often the contraceptive pill, which is composed of synthetic hormones and typically prescribed by health care providers. Our results indicate that users, by displaying a negative narrative around synthetic hormones (similar to participants in the studies by Chen [9] and Grenfell et al [21]), are continuing a trend in public discourse that constructs synthetic hormones as unnatural and, as a consequence, unhealthy. This is in line with the study by Le Guen et al [1], who found a desire for naturalness to be a reason for rejecting hormonal contraception in Western countries. One could speculate that seeing the digital contraceptive as an alternative and being able to choose it on their own might give people a sense of control over their bodies or even of resistance to the medicalization of women’s bodies. However, across themes 1 and 3, we see perspectives agreeing with interviewees in the study by Grenfell et al [21] in that *natural* is compatible with *medical* and *scientific*. Additionally, from our themes and topic 2 (*No side effects contraception*), we see that Natural Cycles, through its use of technology, is also perceived as *modern* and that this is for users a desirable trait, although with some tensions with trustworthiness. Yet, this hesitation in front of new technology (themes 1 and 3) is compensated by users deeming the product both medical and scientific—a conceptualization that the company presents to users early in the user journey as a factor to choose this contraceptive method [81].

Examining further this concept of naturalness of a digital contraceptive, we argue that it is not just a hormone-free naturalness but a *tamed naturalness*—one that is precisely tamed, contained, and regulated by science. Science renders this natural method safe and trustworthy because, for users, science embeds these values—and this link is left unquestioned. Furthermore, our analysis suggests that users do not find a contradiction in conceptualizing the product as natural although the product (an algorithm, a thermometer, and an app) is created, tested, regulated, and maintained by an assemblage of humans, systems, procedures, and institutions (via medical studies, certifications/regulations, and new version releases)—even for users who are aware of these actors taking part in the product’s life cycle, as evidenced by the reviews that mentioned them (theme 1).

In summary, our analysis shows that the tamed naturalness embodied by Natural Cycles can emerge not just as an evidence-based contraceptive approach (which has been scarce in the app landscape [23,24]) but also, at the same time, as a suitable choice for people who, for any reason, opt out of hormone-based methods. Future research on digital contraception could explicitly focus on how users experience tamed naturalness along the user journey and on how to design new digital contraceptives—or interactions with existing digital contraceptives—that enhance this aspect.

### Not Just an Algorithm: An Interactive Agent

From our analysis, and especially from theme 4, we see that Natural Cycles is not just a *digital* contraceptive. A great deal of value is placed by users in the interaction with the app. This interactivity is the basis of mutual learning as the digital contraceptive actively supports learning through visualizations and personalized in-app messages, scaffolding the growth of user expertise along the user journey. By allowing the user to exclude a temperature measurement, the digital contraceptive positions them as an expert in their own data as they learn to identify when they should influence the algorithm. The development of user expertise over time is similar to the findings of Grenfell et al [21] on Plan Pregnancy mode users “doing and seeing the science” and to the observations by French et al [22] about women becoming experts in their own bodies. Our results provide insights on how this phenomenon unfolds in the context of contraception and, in particular, during interactions with the digital contraceptive. Here, it is worth wondering whether users could become so expert that the technology is no longer needed. Cases such as the user who was expecting the digital contraceptive to warn them after inputting that they had had unprotected sex during a red day show that users might still expect to see even more interactivity from the digital contraceptive, seeing it as a source of guidance that could continue to educate them—or even “scold” them [8]. Future research could explore appropriate interactions (including their timing and ethical implications) for the digital contraceptive to also play a “warning” or “scolding” role.

Moreover, our analysis shows that who gets to be framed as the expert is situated and part of a negotiation between the user and the technology that unfolds when, for instance, the predictions do not match the user’s embodied knowledge and in which partners and health care professionals have a role to play (themes 4 and 5). For example, although some users were confident in reading their own body, they still used the predictions to confirm their own feelings (ie, the technology remained integral to them trusting their self-knowledge, adding to previous arguments [82-84] about self-tracking). Specifically, the role of the digital contraceptive as an algorithmic mediator has to do with the perceived objectivity of the algorithmic data over the subjective nature of the user’s embodied experiences [85]. Although also compatible with the finding by Grenfell et al [21] on Natural Cycles privileging disembodied data, our work adds nuance as topics 8, *Body or algorithm*, and 9, *(Dis)trust in BBT method*, show that some users have learned when to trust their own bodies, similar to participants in the study by Algera [26] appropriating menstrual and fertility trackers for contraception and to the analysis by Park et al [8] of ambivalences in digital contraception. Some partners apparently also needed the confirmation provided by the technology (theme 5). This resonates with our previous findings [86] about physicians disregarding women’s accounts and concerns about their perimenopause until hormonal test results came back “confirming” what patients already knew and with the reports by French et al [22] on women being dismissed by health care professionals regarding their fertility concerns. This opens a potential avenue for future research to study matters of trust among relevant actors (primary users, partners, health care professionals, and the digital contraceptive itself) and, in particular, how trust is achieved, negotiated, or lost in interactions with the digital contraceptive.

### Medically Noninvasive: Intimately and Socially Intervening

Our analysis adds to findings on self-tracking supporting people in achieving their personal goals and making them feel more in control of their own lives [87]. Theme 2 captures how users frame their cultivation of self-knowledge as a means toward self-control and this, explicitly, as empowering—at a personal level and potentially at the societal level.

Building on our analysis, we argue that the concept of “proper use” that users refer to (theme 3) contributes to constructing an idea of the *good user*, the one who *does their part* to help the algorithm with the promise of empowerment. Moreover, our analysis highlighted user diligence and the need for an organized life as core to the user experience as the number of green days increases with frequent, good-quality data. This suggests that this digital approach to contraception continues to position the menstruating body as something to be controlled. Users seem to feel that, to rule their bodies, they should manage the practical aspects of their life—avoiding irregular sleeping times and the excess of alcohol, adhering to a particular morning routine (which generates frustration in some partners), explaining their feelings and experiences, and scheduling activities around their cyclical changes (themes 3 and 5). The responsibility and additional burden of managing a lifestyle is shared by those using fertility tools for conception [88] but seems to constitute new work for those in need of complying with a digital contraceptive. Furthermore, reviews that enthusiastically stated that the BBT method should be taught in schools referred to “girls,” which signals a societal view in which the responsibility of contraception (including emotional and practical labor) is still largely placed on women (with nonwomen menstruators absent in this discourse). In spite of this, our analysis also highlights how this self-tracking device can make users feel empowered—an empowerment so important that, for some, it compensates for the burden of one-sided reproductive responsibilities.

Our analysis also identified empirical details about how couples collaborate in the use of Natural Cycles as a contraceptive. Theme 5 captures stories of partners as collaborators, becoming actively involved in the process as well as explicitly “sharing the burden” and learning about fertility and menstrual cycles. We highlight how people manage contraception and its interpersonal, collaborative aspects through the digital contraceptive—as well as how some become aware of their one-sided efforts. This adds to the results of Grenfell et al [21], where the involvement of partners in planning a pregnancy was limited to taking care of data input or becoming interested only because of the statistics or mistrust in the method. This is interesting given that planning a pregnancy is likely to span a shorter period than digital contraception—which can last potentially for decades. It is important to complicate this scenario by considering the cases in which users needed to show their Natural Cycles data for their partners to confirm what was happening as this suggests that the feeling of empowerment could be removed in seemingly simple interactions within the couple—in line with the findings of Park et al [8], where some participants perceived partner involvement as an unwelcome interference. Taken together, this all suggests that future research on digital contraceptives should look at how to adequately involve partners in the use of digital contraception when this is wanted by the primary user.

Finally, our analysis (and especially theme 3) found that the app’s design and its business model suggest certain types of users and data, with practical requirements including financial situation (to keep the subscription renewed), sleeping patterns (to be able to produce good-quality data frequently), and cycle regularity (for the algorithm to grant green days). Regarding the latter, Natural Cycles states that the product is “just as effective” but “might be less suitable” for people with irregular cycles [30] as they will see more red days, which the company acknowledges as a factor for reduced user satisfaction [89]—contributing to show that the user experience of a digital contraceptive complements its medical effectiveness. This all leads to the question of *who* can make “proper use” of this digital contraceptive or, in other words, *who* the digital contraceptive is designed for. This resonates with the critique by Oudshoorn [90] of the one-size-fits-all approach in the design of contraceptive technologies such as the pill with no regard to diversity among consumers*.* It also becomes particularly relevant as large-scale quantitative studies about “the menstrual cycle” are being run using data from Natural Cycles users while intending to shed light on the general population’s menstrual cycle characteristics (eg, the study by Bull et al [36] only mentions lower obesity when comparing participants with women in the general population). Future research could target the user experience of people with irregular cycles as a first step toward more inclusive digital contraception.

### Limitations

As the Google Play Store does not associate geographic or demographic data with reviews, we were not able to know the users’ location, level of income, gender, or mother tongue. Having these variables to contextualize their experiences could have allowed us to, for example, apply an ecological perspective and sociocultural lenses, similar to the studies by Figueiredo and Chen [20] and Bhat and Kumar [91]. Although a feminist approach to data science implies preserving the data’s context [49,74], we recognize a fundamental tension with user privacy, which reinforced our will to not collect more detailed data about users and even to reduce the precision of our data set by not storing any identifying information. On the basis of English-language reviews, our work continues to amplify a certain kind of voice. Adapting our pipeline to work with other languages could be a starting point to increase diversity. In addition, our data set does not include iPhone users’ reviews owing to a restriction imposed by the Apple App Store under which only the last 50 reviews of an app could be fetched. Finally, there are limitations to using reviews as a source of knowledge about experience. App reviews may be manipulated to skew the perception of a company or tool. For example, companies may attempt to flood a site with positive reviews. However, Simonson and Rosen [38] argue that these behaviors are easily spotted by customers and are not worth the loss in reputation if discovered, and Google Play has a range of fraud detection mechanisms even if these do not always deter fraudulent reviews [92]. In addition, users who are particularly happy or unhappy with a product might be more motivated to leave a review than those who feel more neutral toward it, which could bias a review data set toward extreme experiences. Still, reviews with middle-range ratings (2-4 stars) represented 17.7% (578/3265) of the data set, which is not a negligible portion. On balance, although we do not suggest that this should be the only approach to understanding the experience of a digital contraceptive, we believe that it offers a useful perspective on these technologies.

### Conclusions

Our mixed methods analysis of user reviews of Natural Cycles, the first digital contraceptive, contributes an examination of how users experience digital contraception. Through the concept of *tamed naturalness*, we articulate how the science and technology of a digital contraceptive are perceived by users as offering an evidence-based type of natural contraception. We show how the interactivity of the digital contraceptive via personalized in-app messages is core to the user experience, providing motivation to keep going and educational content and supporting expertise along the user journey. We highlight the labor entailed in managing oneself to provide accurate and frequent data for the digital contraceptive and point out the exclusions that occur when user diligence is not enough. We temper this with the opportunity that digital contraceptives provide for some users to initiate intimate collaboration around contraceptive practices and responsibilities.

Given that people are increasingly turning to “natural” contraception approaches and that our work shows that individuals can conceptualize a method based on a digital product as natural, we argue that digital technology will increasingly have a role to play in the space of contraception. Building on our analysis, future research and innovation could look at designing or redesigning digital contraceptives with primary users and intimate partners, enhancing the experience of tamed naturalness; exploring how trust fluctuates among involved actors and in interactions with the technology; and, ultimately, devising more inclusive approaches to digital contraception.

## Abbreviations

BBT: basal body temperature
LDA: latent Dirichlet allocation

## Data Availability

The data set analyzed during this study is available from the corresponding author upon reasonable request.
